# The p75 Neurotrophin Receptor Is a Central Regulator of Glioma Invasion 

**DOI:** 10.1371/journal.pbio.0050212

**Published:** 2007-08-14

**Authors:** Angela L. M Johnston, Xueqing Lun, Jennifer J Rahn, Abdelhamid Liacini, Limei Wang, Mark G Hamilton, Ian F Parney, Barbara L Hempstead, Stephen M Robbins, Peter A Forsyth, Donna L Senger

**Affiliations:** 1 Department of Biochemistry and Molecular Biology, University of Calgary, Calgary, Alberta, Canada; 2 Southern Alberta Cancer Research Institute, Calgary, Alberta, Canada; 3 Clark H. Smith Integrative Brain Tumour Research Center, Calgary, Alberta, Canada; 4 Department of Oncology, University of Calgary, Calgary, Alberta, Canada; 5 Department of Clinical Neurosciences, University of Calgary, Calgary, Alberta, Canada; 6 Division of Hematology, Cornell University Medical College, New York, New York, United States of America; Fred Hutchinson Cancer Research Center, United States of America

## Abstract

The invasive nature of cancers in general, and malignant gliomas in particular, is a major clinical problem rendering tumors incurable by conventional therapies. Using a novel invasive glioma mouse model established by serial in vivo selection, we identified the p75 neurotrophin receptor (p75^NTR^) as a critical regulator of glioma invasion. Through a series of functional, biochemical, and clinical studies, we found that p75^NTR^ dramatically enhanced migration and invasion of genetically distinct glioma and frequently exhibited robust expression in highly invasive glioblastoma patient specimens. Moreover, we found that p75^NTR^-mediated invasion was neurotrophin dependent, resulting in the activation of downstream pathways and producing striking cytoskeletal changes of the invading cells. These results provide the first evidence for p75^NTR^ as a major contributor to the highly invasive nature of malignant gliomas and identify a novel therapeutic target.

## Author Summary

Gliomas are highly malignant and invasive tumors with tendrils that extend far from the primary tumor site, rendering conventional therapies ineffective and leading to an invariably poor prognosis. To understand the molecular mechanisms underlying this invasive behavior, we injected immunocompromised mice with human gliomas and compared invasive cells, which left the primary tumor site, to noninvasive cells, which remained at the site of injection. We identified the neurotrophin receptor p75^NTR^—which normally functions during development to induce neurite outgrowth and promote neuronal cell death—as an important regulator of glioma invasion. We present the first evidence that this neurotrophin receptor can also be a potent mediator of glioma invasion, and we show that the expression of this receptor is sufficient to impart a dramatic invasive behavior on genetically distinct tumors. These data highlight a previously unknown function of this receptor and suggest it may be a novel therapeutic target in the treatment of this devastating cancer.

## Abstract 3

By in vivo selection of a human glioma, the authors identify the p75^NTR^ neuotrophin receptor as a critical molecule regulating increased invasiveness.

## Introduction

Malignant gliomas are diffuse, highly invasive, and often multifocal tumors that have a dismal prognosis, with a median survival of only 1 y and “long-term survivors” (i.e., surviving ≥3 y) are rare [1,2]. A major barrier to effective malignant glioma treatment is their highly invasive nature; they extend tendrils of tumor several centimeters away from the main tumor mass, which render these tumors incurable by local therapies such as surgery or radiotherapy [3]. Ninety-five percent of gliomas recur within 2.5 cm of the resection margin, which contains invasive cells that act as a “disease reservoir” and elude current treatments [4]. Glioma cells do this by becoming distinct from their noninvasive counterparts. Specifically, they activate a number of coordinate cellular programs, which include those necessary for migration (e.g., motility) and invasion (e.g., extracellular matrix degradation) [5] and also a number of pathways (e.g., reduced proliferation, marked resistance to apoptosis) [4,6,7] which render the invasive cells highly resistant to conventional treatments. A detailed understanding of the mechanisms underlying this invasive behavior is essential for the development of effective therapies.

Although in their infancy, attempts to identify genes involved in glioma invasion have used a number of techniques, including the isolation of invasive cells from human cell lines in vitro [6,7], the use of organotypic brain slice cultures [8], and the collection of tumor and invasive cells from frozen glioblastoma patient specimens using laser capture microdissection [9–11]. Although each method has been successful in its own right, none of these models have been ideal or comprehensive for discovering the underlying mechanisms of invasion. New models or alternative strategies are needed. We have therefore undertaken the approach of serial in vivo selection to identify genes important for the invasive behavior of malignant glioma. Similar strategies have been used to effectively identify mechanisms underlying the metastatic behavior of both melanoma and breast tumors [12,13]. Using this approach, we isolated highly invasive glioma cells from a relatively noninvasive human malignant glioma. Gene expression profiles comparing these two tumor cell populations identified the p75 neurotrophin receptor (p75^NTR^) as an important and potent mediator of invasion in human glioma.

p75^NTR^ is a transmembrane glycoprotein and a member of the tumor necrosis factor (TNF) superfamily that was originally isolated as a nerve growth factor (NGF) receptor, but has since been shown to bind both the mature and precursor forms of the neurotrophin family of ligands (brain-derived neurotrophin factor [BDNF], neurotrophin-3 [NT-3], and neurotrophin-4/5 [NT-4/5]) [14–18]. In neurons, p75^NTR^ is coexpressed with a second group of neurotrophin receptors, the tropomysin receptor kinases (Trks). It has become increasingly clear that the dogma in neuroscience that Trks mediate neuronal survival and p75^NTR^ causes neuronal cell death is too narrow a view [19–22]. Rather, there is a growing appreciation that p75^NTR^, like other members in the TNF superfamily, mediates a very broad range of cellular functions, depending on the cell context and the repertoire of co-receptors that exist (e.g., Trks [23], Nogo receptor [24], and sortilin [25]). In neurons, p75^NTR^ has been shown to increase [26,27] or inhibit [28] axon growth, reduce [29] or promote [30,31] neuronal cell death, and is either necessary [32] or not required [33] for inhibition of neuronal regeneration. These apparent discrepancies are not confined to neurons; p75^NTR^ has also been shown to both inhibit [34] and promote [26,35] Schwann cell migration during development. Even though p75^NTR^ does not contain any catalytic activity, it interacts with several proteins that help transmit signals required for its various functions. Neurotrophin engagement of p75^NTR^ controls the activity of the small GTPase RhoA, providing a direct link from the receptor to modulating cellular architecture. As is the case for phenotypic responses, RhoA has been shown to be activated or inhibited depending on cellular context [27,28,34,36–38]. Reports have hinted at roles for p75^NTR^ in growth [39] and apoptosis [40] of glioma cells; however, data presented here support a much different role for p75^NTR^—that of mediating glioma cell invasion.

## Results

### Establishment of a Malignant Glioma Mouse Model to Study the Molecular Determinants of Glioma Invasion

**Figure 1:**
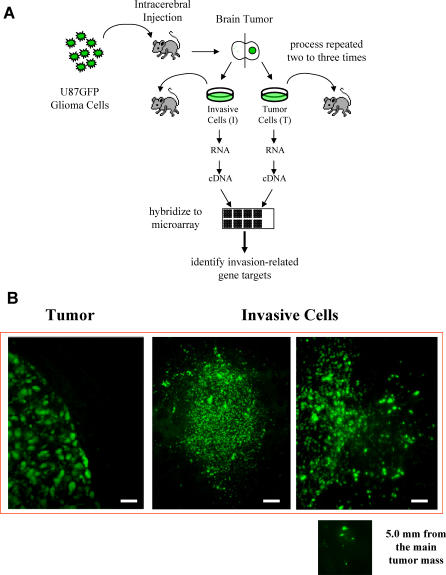
Serial In Vivo Selection Was Used to Isolate a Highly Invasive Glioma Population from a Noninvasive Human Malignant Glioma Cell Line (A) The noninvasive human glioma cell line U87 stably expressing GFP (U87GFP) was implanted into the brains of SCID mice. Four to 6 wk later, the mice were sacrificed. The ipsilateral side of the brain (containing a grossly visible tumor) was separated from the contralateral side (containing only isolated invasive glioma cells [i.e., no macroscopically visible GFP-labeled tumor]), and both were grown in culture. These noninvasive (tumor) and highly invasive glioma cells were reimplanted into additional mice, and the process was repeated to select for increasingly noninvasive or invasive glioma cells. RNA extracted from the resulting invasive and tumor populations was used to prepare labeled cDNA that was hybridized to oligonucleotide microarrays. (B) Brains of SCID mice implanted with either tumor (left) or invasive (center and right) glioma cells. GFP visualization reveal the well-circumscribed border of the reimplanted tumor cells, with no tumor cells being detected away from the main tumor mass (left). This is in sharp contrast to the highly invasive border of the invasive tumors, where isolated small groups of glioma cells are found throughout the brain (center and right). Scale bars on GFP images represent 125 μm (center) and 62 μm (right and left).

**Figure 2:**
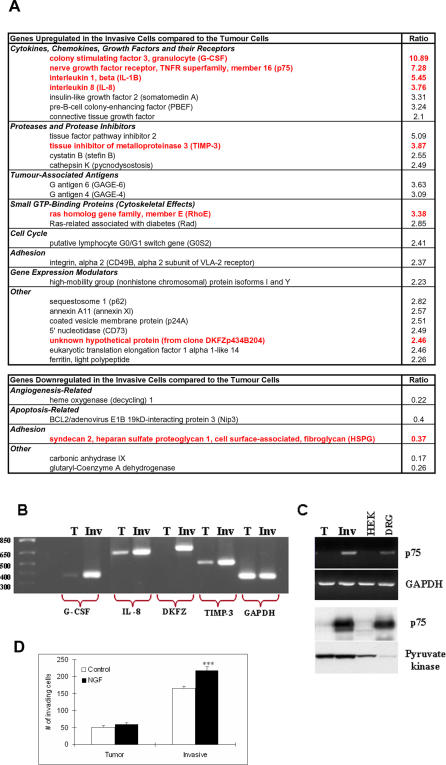
Microarray Experiments Were Performed to Compare the Gene Expression Differences between the In Vivo–Selected Noninvasive (Tumor) and Invasive Glioma Cells (A) Table lists results from a representative set of lineage experiments. Four independent microarray experiments were performed, each containing a pair of dye-flipped hybridizations. Genes that displayed consistent gene expression changes (>2-fold change in at least five out of eight hybridizations) are listed. The indicated ratios represent the fold change in gene expression in the invasive compared to the noninvasive cells. Genes chosen for validation are indicated in red. (B and C) Seven genes were chosen for validation; the expression of five are shown. (B) RT-PCR confirmed the expression of granulocyte colony-stimulating factor (G-CSF), interleukin-8 (IL-8), an unknown hypothetical protein DZFKp434B204 (DZFK), and tissue inhibitor of metalloproteinases-3 (TIMP-3) in the invasive population. Expression levels of GAPDH (unchanged) are shown for comparison. (C) RT-PCR and Western blot confirm expression of p75 in the invasive population (Inv) but not the tumor cell population (T). RT-PCR analysis of GAPDH levels and Western blot analysis of pyruvate kinase levels are included as loading controls. Human dorsal root ganglia (DRG) were used as a positive control. (D) Addition of NGF (200 ng/ml) enhanced the migratory ability of the invasive glioma cells in matrigel-coated invasion chambers, but had no significant effect on invasion of the tumor cells. Values shown are the mean ± SEM from three independent experiments. Triple asterisks (***) indicate *p* < 0.001 versus control (two-way ANOVA with Bonferroni post-tests).

One of the problems in xenotransplanting human glioma cells into the brains of immunocompromised mice is that the resulting tumors are circumscribed, with very little cell infiltration into the brain parenchyma [41]. To generate an orthotopic model that more closely mimics the human disease and allows for the identification of molecular determinants of glioma invasion in a global and unbiased manner, we used an in vivo–selection procedure to select for highly invasive human glioma cells (Figure 1A). We isolated highly invasive glioma cells from the noninvasive human malignant glioma cell line U87 expressing green fluorescent protein (GFP) (U87GFP) and a neomycin resistance gene. Expression of these genes afforded us the ability to isolate the rare glioma cell that migrated away from the primary tumor site. These “invasive” cells were grown and expanded in tissue culture, and reintroduced into the brains of immunocompromised mice where they formed highly infiltrative tumors with poorly defined edges (Figure 1B). These extremely invasive cells were found vast distances from the main tumor mass, with GFP-positive tumor cells readily identifiable in the contralateral hemisphere. In clear contrast, reimplantation of the noninvasive “tumor” cells led to the formation of large tumors with sharply demarcated edges (Figure 1B). Using this model, we identified gene expression differences between the noninvasive and highly invasive in vivo–selected glioma cells. RNA extracted from tumor and invasive populations was used to prepare labeled cDNA that was hybridized to 14,000-gene human oligonucleotide microarrays (produced by the Southern Alberta Microarray Facility, University of Calgary). Genes up- or down-regulated in the invasive population were compared to the tumor population, and genes that showed consistent gene expression changes of 2-fold or greater are outlined in Figure 2A. To ensure the integrity of the microarray data, we chose seven arbitrary genes for validation, the expression of five of which are shown in Figure 2B and 2C. Semiquantitative real-time polymerase chain reaction (RT-PCR) confirmed the expression of all seven genes, including granulocyte colony-stimulating factor (G-CSF), interleukin-8 (IL-8), DZFKp434B204 (unknown hypothetical protein), tissue inhibitor of metalloproteinases-3 (TIMP-3), and p75^NTR^ (Figure 2B and 2C). The semiquantitative RT-PCR indicates that our microarray data is an under-representation of the fold changes in RNA expression. Based on the reproducibility of the data, previous implication in tumorigenesis in other cancers (e.g., melanoma and prostate) [42–47], and the novelty of the finding in brain tumors, we chose p75^NTR^ for further study. Importantly, we confirmed the up-regulation of p75^NTR^ was not only at the mRNA level, but that a dramatic alteration in abundance of p75^NTR^ was seen in the invading cells (Figure 2C). A number of invasive lines were generated by serial in vivo selection and microarray analysis using a second independent U87 invasive line validated the presence of p75^NTR^ by microarray that was confirmed by RT-PCR and Western blot (unpublished data). In addition, using the in vivo–selection paradigm outlined in Figure 1, we isolated both tumor and invasive cells from a second human glioma cell line, U251N. These in vivo–selected invasive U251N cells also expressed high levels of endogenous p75^NTR^ (Figure S1).

### In Vivo Selection Identifies p75^NTR^ as a Mediator of Glioma Invasion

Although p75^NTR^ and its ligands, the neurotrophins, are expressed throughout the nervous system, particularly during development, a role for p75^NTR^ in central nervous system tumors has not been described previously. We therefore assessed whether the up-regulation of p75^NTR^ found in the invasive glioma cells had a functional consequence (i.e., increased their migration and invasion). The noninvasive and highly invasive cells were treated with the p75^NTR^ ligand NGF, and migration and invasion were measured. The addition of NGF to invasive cells significantly increased the number of cells able to invade through matrigel, but had no effect on the invasive ability of the tumor cells (which had no detectable p75^NTR^; Figure 2D). Because neurotrophins are also ligands for the Trk receptors, RT-PCR and immunoprecipitation experiments were performed. No detectable mRNA or protein for the Trk receptors was found in the invading glioma cells (unpublished data). In addition, we tested the effect of the unprocessed or proform of NGF (pro-NGF), a high-affinity ligand for p75^NTR^ [33,39] that is unable to activate Trk [16]. Accordingly, treatment of the invasive cells with cleavage-resistant pro-NGF enhanced their migration at concentrations as low as 1 ng/ml while having no effect on the tumor cells (Figure S2). Although we found that neurotrophin could enhance invasion of the p75^NTR^-positive invasive cells (Figure 2D), we also observed a significant increase in the absence of ligand. Signals from p75^NTR^ can arise both in the absence and presence of ligand; however, these signals often evoke opposing biological responses. Because the outcome of both neurotrophin-dependent and neurotrophin-independent signaling was the same, we considered the possibility that the glioma cells were producing and secreting neurotrophin(s), thus activating an autocrine loop. We assessed the expression of several neurotrophins and found that BDNF was present in both the conditioned media and the cell lysate of all glioma cells tested (unpublished data). Furthermore, we found that the presence of p75^NTR^ shifted the localization of BDNF from the conditioned media to the cell membrane (Figure S3), supporting the notion of autocrine/paracrine activation of the p75^NTR^ receptor.

**Figure 3:**
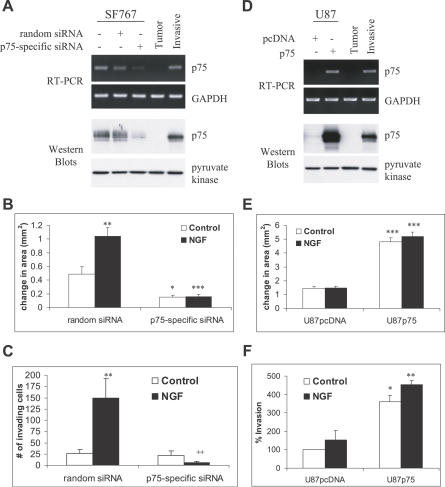
p75^NTR^ Induces Migration and Invasion In Vitro (A) Down-regulation of p75^NTR^ using RNAi decreases glioma migration/invasion. RT-PCR (GAPDH used as a loading control) and Western blot (pyruvate kinase used as a loading control) for p75^NTR^ confirm down-regulation of p75^NTR^ in the glioma cell line SF767 transfected with a p75-specific siRNA. Untransfected cells, cells transfected with a random siRNA, and the in vivo–selected tumor and invasive cells are shown for comparison. (B) Treatment with NGF (200 ng/ml) enhanced migration of SF767 cells transfected with the random (control) siRNA, but had no significant effect on migration of SF767 cells in which p75^NTR^ expression was inhibited by p75^NTR^-specific siRNA. Values shown are the mean ± SEM from three independent experiments. A single asterisk (*) indicates *p* < 0.05, and double asterisks (**) indicate *p* < 0.01 versus control-treated random siRNA-transfected cells; triple asterisks (***) indicate *p* < 0.001 versus NGF-treated random siRNA-transfected cells (two-way ANOVA with Bonferroni post-tests). (C) Treatment with NGF (200 ng/ml) enhanced invasion of SF767 cells transfected with random siRNA, but had no significant effect on invasion of SF767 cells in which p75^NTR^ expression was inhibited by p75^NTR^-specific siRNA. Values shown are the mean ± SEM from a single experiment. Similar results were seen in two independent experiments. Double asterisks (**) indicate *p* < 0.01 versus control-treated random siRNA-transfected cells, and double pluses (++) indicate *p* < 0.01 versus NGF-treated random siRNA-transfected cells (two-way ANOVA with Bonferroni post-tests). (D) Ectopic expression of p75^NTR^ induces glioma migration/invasion. RT-PCR (GAPDH used as a loading control) and Western blot (pyruvate kinase used as a loading control) for p75^NTR^ confirm expression of p75^NTR^ in U87 cells stably transfected with pcDNA3.1 encoding human p75^NTR^ (U87p75). Cells stably transfected with the empty pcDNA3.1 vector (U87pcDNA), as well as in vivo–selected tumor and invasive cells are shown for comparison. (E) Migration of U87 glioma cells is enhanced by ectopic expression of p75^NTR^. No additional increase was seen following treatment with NGF (200 ng/ml). Values shown are the mean ± SEM from three independent experiments. Triple asterisks (***) indicate *p* < 0.001 versus pcDNA-transfected cells (two-way ANOVA with Bonferroni post-tests). (F) Similarly, invasion of U87p75 glioma cells in matrigel-coated invasion chambers was significantly increased compared to controls. No further increase was seen with exogenous NGF (200 ng/ml). Values shown are the mean ± SEM from four independent experiments. A single asterisk (*) indicates *p* < 0.05, and double asterisks (**) indicate *p* < 0.01 versus pcDNA-transfected cells (two-way ANOVA with Bonferroni post-tests).

To directly test the hypothesis that elevated expression of p75^NTR^ is necessary for neurotrophin-induced glioma migration and invasion, we surveyed a panel of human glioma cell lines for p75^NTR^ protein expression. We found that the human glioma cell line SF767 endogenously expressed high levels of p75^NTR^, as detected by Western blot (Figure 3A) and fluorescence-activated cell-sorting (FACS) analysis (unpublished data). Using RNA interference (RNAi), we down-regulated p75^NTR^ in the SF767 cell line using an expression vector containing a p75^NTR^-specific small interfering ribonucleic acid (siRNA) and confirmed the down-regulation by RT-PCR and Western blot (Figure 3A). A random, nonspecific siRNA sequence was used as a control. Down-regulation of p75^NTR^ levels in SF767 was sufficient to reduce its migration in vitro and rendered the cells nonresponsive to addition of NGF in both migration and invasion assays (Figure 3B and 3C). Similarly, down-regulation of p75^NTR^ by siRNA in the original in vivo–selected U87 invasive cells significantly blocked migration and invasion (Figure S4).

Since down-regulation of p75^NTR^ in SF767 cells and U87 in vivo–selected invasive cells inhibited glioma invasion, we assessed whether ectopic expression of p75^NTR^ alone was sufficient to increase glioma migration and invasion in a cell line without detectable p75^NTR^ (the original U87 cell line). To this end, we stably transfected the full-length cDNA of human p75^NTR^ into the U87 glioma cell line, using stable transfection of the empty pcDNA vector as a control. Expression levels of p75^NTR^ in these cells were confirmed by RT-PCR and Western blot (Figure 3D). Expression of p75^NTR^ caused a significant increase in migration and invasion in vitro (Figure 3E and 3F). Treatment of these cells with NGF had no further enhancement on their migration or invasion consistent with the idea that when p75^NTR^ is expressed, an autocrine loop is completed, leading to enhanced migration and invasion.

### Expression of p75^NTR^ Increases Invasion In Vivo in Genetically Distinct Glioblastomas

**Figure 4:**
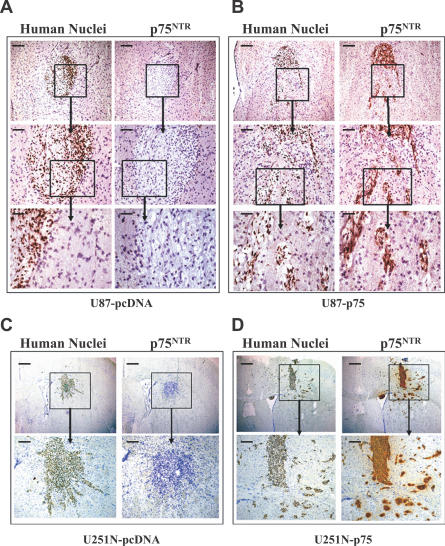
Expression of p75^NTR^ in the U87 and U251N Glioma Cell Lines Dramatically Increases Invasion In Vivo U87 or U251N human glioma cells stably transfected with the empty pcDNA vector (U87pcDNA [A] or U251NpcDNA [C]) or the p75^NTR^-expression vector (U87p75 [B] or U251NpcDNA [D]) were implanted into the brains of SCID mice and allowed to grow for 28 d. The mice were sacrificed, and frozen brain sections were stained with antibodies against human nuclei (left) and human p75^NTR^ (right). Boxed areas indicate the region shown in the panel below, thus magnification increases from top to bottom; scale bars in (A) and (B) represent 100 μm, 50 μm, and 25 μm; and scale bars in (C) and (D) represent 200 μm and 100 μm. Implantation of U87 glioma cells stably transfected with the empty pcDNA vector led to the formation of well-circumscribed tumors that were p75^NTR^ negative (A). In sharp contrast, implantation of U87 glioma cells ectopically expressing p75^NTR^ led to the formation of tumors with highly infiltrative edges (B). Similar results were seen in three independent experiments with six animals in each group. U251NpcDNA glioma cells were generally more invasive than U87pcDNA cells upon implantation into the brains of SCID mice, and formed tumors with finger-like projections extending into the surrounding normal brain (C); nevertheless, ectopic expression of p75^NTR^ in U251N cells dramatically increased the invasiveness of these cells in vivo with isolated individual tumor cells being found distant from the main tumor mass (D). Similar results were seen in all ten animals in each group.

Malignant gliomas clinically show extensive infiltration away from the main tumor and into the surrounding normal brain tissue. To determine whether the expression of p75^NTR^ was important for glioma cell invasion in vivo, we implanted the U87 human glioma cell line ectopically expressing p75^NTR^ into the brains of severe combined immunodeficiency (SCID) mice. U87 cells stably transfected with the empty pcDNA vector were implanted for comparison as a control. Twenty-eight days after implantation, the mice were sacrificed and the brains prepared for immunohistochemical staining using antibodies directed against human nuclei and p75^NTR^. Implantation of U87 glioma cells stably transfected with pcDNA led to the formation of well-circumscribed tumors that were p75^NTR^ negative (Figure 4A). In sharp contrast, implantation of U87 glioma cells stably expressing p75^NTR^ resulted in the formation of tumors with highly infiltrative edges (Figure 4B). Isolated p75^NTR^-positive human glioma cells could be detected in regions vastly distant from the main tumor mass (Figure S5).

Because malignant gliomas are an extremely heterogeneous group of tumors and the in vivo–selected U251N cells also expressed high levels of p75 (Figure S1), we determined whether the sole expression of p75^NTR^ was sufficient to impart an invasive phenotype, not only on the U87 cells, but also on the genetically distinct U251N cells. U251N cells ectopically expressing p75^NTR^ (U251Np75), along with empty vector–transfected cells as a control (U251NpcDNA), were implanted into the brains of SCID mice as described above. As we have observed previously, the U251N control cell line (U251NpcDNA) was inherently more invasive than U87pcDNA in vivo, with finger-like projections extending from the main tumor mass into the surrounding normal brain (compare Figure 4A and 4C). Nevertheless, ectopic expression of p75^NTR^ (U251Np75) dramatically enhanced the inherent invasive ability, with p75^NTR^-positive cells being found at locations distinct from the main tumor mass (compare Figure 4C and 4D). Thus, up-regulation of p75^NTR^ is sufficient to allow glioma cells of diverse genetic backgrounds to invade into the surrounding normal brain. Because p75^NTR^ can have effects on several physiological responses, we also evaluated the effect of p75^NTR^ expression on cell cycle, proliferation, and survival, and observed no significant change (unpublished data).

### p75^NTR^-Mediated Glioma Invasion Is Neurotrophin Dependent

**Figure 5:**
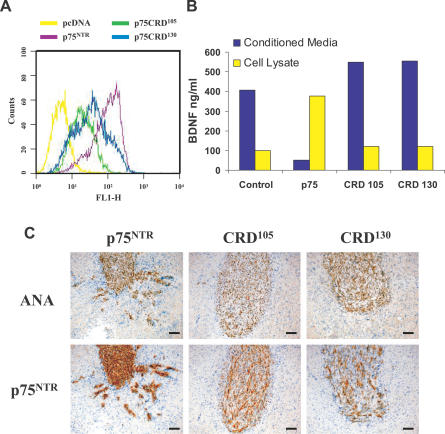
p75^NTR^-Induced Glioma Invasion Is Neurotrophin Dependent (A) U87 human glioma cells stably transfected with wild-type p75^NTR^ (red), the neurotrophin binding mutant p75CRD^105^ (green), and p75CRD^130^ (blue) or pcDNA empty vector (yellow) were examined for cell surface expression of p75 receptors by flow cytometry using the p75-specific primary antibody and an Alexa 448–conjugated secondary antibody. (B) Expression of BDNF in the conditioned medium (blue bar) and the total cell lysate (yellow bar) of U87 glioma cells expressing pcDNA (control), p75^NTR^ (p75), CRD 105, and CRD 130 were analyzed by ELISA. BDNF was found in the cell-associated fraction of U87 cells expressing p75, but not in the cells expressing the neurotrophin binding mutants *CRD^105^* or *CRD^130^*. (C) U87 cells expressing wild-type p75^NTR^, neurotrophin binding mutant *CRD^105^,* or *CRD^130^* were implanted into the brains of SCID mice and allowed to grow for 28 d. The mice were sacrificed, and frozen brain sections were stained with antibodies against human nuclei (ANA; top row) and human p75^NTR^ (bottom row). Scale bars in (C) represent 100 μm. Implantation of either U87 glioma cells stably transfected with *CRD^130^* or *CRD^105^* vector, in contrast to the highly infiltrative edges of U87p75, led to the formation of well-circumscribed tumors. Similar results were seen in three independent experiments with six animals in each group. Expression of neurotrophin binding mutants of p75^NTR^ negates p75-induced glioma invasion.

In order to test whether neurotrophin was important in the invasive behavior of these cells, we constructed two p75^NTR^ mutants, *p75CRD^105^* and *p75CRD^130^,* containing a four–amino acid insertion in the cysteine-rich domain (CRD) following amino acids 105 and 130 (CRD 105 and CRD 130), respectively. Insertions at these locations disrupt the normal spacing of the cysteine residues within the164 ligand-binding domain and create p75^NTR^ proteins that are unable to bind to mature neurotrophin [48]. These constructs were stably transfected into U87 glioma cells, and cell surface expression for the mutant p75^NTR^ proteins was confirmed by FACS analysis (Figure 5A). To verify that the mutant p75^NTR^ do not bind neurotrophin, BDNF expression in the conditioned medium and total cell lysates of U87 cells expressing *CRD^105^* and *CRD^130^* were performed. Unlike the wild-type p75^NTR^-expressing glioma cells in which expression of p75^NTR^ causes a shift in BDNF localization from the medium to the cell lysate, cells expressing the mutant alleles *(CRD^105^* and *CRD^130^)* did not result in a shift of BDNF localization, confirming that these mutants do not bind endogenous BDNF (Figure 5B). These cells were implanted into the brains of SCID mice and allowed to grow for 21 d. The mice were sacrificed, and frozen brain sections were stained with antibodies against human nuclei (Figure 5C; brown color, top row) and human p75^NTR^ (Figure 5C; brown color, bottom row). Disruption of the neurotrophin binding capacity of p75^NTR^ results in tumors with well-defined borders similar to tumors formed by the parental U87 glioma cells that do not express p75^NTR^. These data suggest that neurotrophin binding is required for p75^NTR^-mediated glioma invasion.

### p75^NTR^-Expressing Glioma Cells from Human Glioma Surgical Specimens Show Enhanced Migration

**Figure 6:**
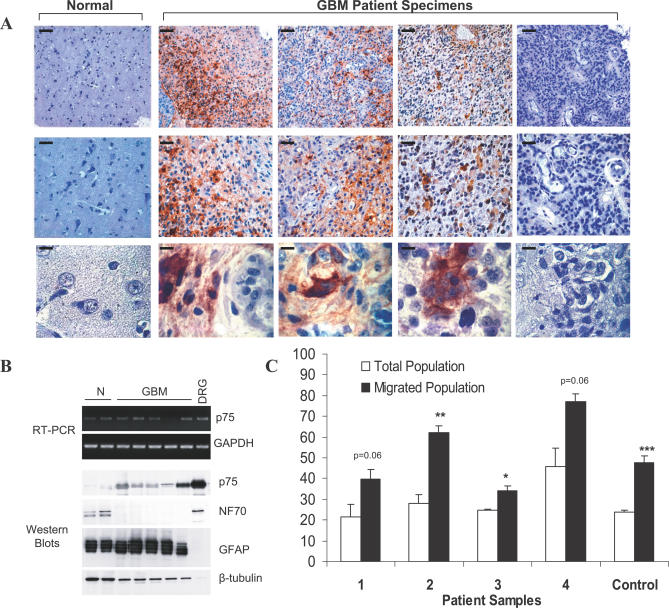
p75^NTR^ Is Present and Confers Increased Migratory Ability in Glioblastoma Multiforme Patient Specimens (A) Expression of p75^NTR^ (brown) was examined by immunohistochemistry in normal human brain specimens (zero of five) and GBM patient specimens (17 of 20). Representative samples are shown. Slides were counterstained with hematoxylin (blue). Scale bars from top to bottom represent 100 μm, 50 μm, and 25 μm. (B) p75^NTR^ mRNA and protein were assessed in glioblastoma patient specimens (GBM) and normal human brain (N). Human dorsal root ganglia (DRG) were used as a positive control. GAPDH was used as an internal loading control for RT-PCR. β-Tubulin was used as a protein loading control. Neuronal (neurofilament 70: [NF70]) and glial (glial fibrillary acid protein [GFAP]) markers in patient specimens are shown. (C) p75^NTR^-positive glioma cells from patient specimens have an increased migratory ability. Migration of in vitro–cultured glioma patient specimens was evaluated using transwell motility assays. As a positive control for the assay, a mixture of 25% U87p75 cells and 75% U87pcDNA cells were analyzed at the same time as the patient samples. For both the control sample and the patient samples, the percentage of p75^NTR^-positive cells in the migratory population (cells that migrated to the underside of the transwell) was increased compared to the percentage of p75^NTR^-positive cells in the original population. Values shown are the mean ± SEM. A single asterisk (*) indicates *p* < 0.05; double asterisks (**) indicate *p* < 0.01; and triple asterisks (***) indicate *p* < 0.001 (*t*-test within a given sample).

The highly invasive nature of malignant gliomas has been a substantial barrier in the treatment of patients with this disease. Data presented here strongly suggest that p75^NTR^, in a neurotrophin-dependent manner, is an important regulator of glioma invasion. To clinically validate p75^NTR^'s role in glioma migration and invasion, and demonstrate its relevance in malignant glioma patient specimens, we analyzed the expression of p75^NTR^ in a panel of surgically resected tumor specimens and normal human brain using immunohistochemical staining (Figure 6A), RT-PCR, and Western blot (Figure 6B). Expression of p75^NTR^ protein was detected in 20 of 40 human glioma patient specimens (50%) (one of 11 low-grade astrocytomas [8%], two of nine mid-grade astrocytomas [22%], and 17 of 20 glioblastoma multiforme (GBM) specimens [85%]) and was undetectable in normal human brain (zero of five). Thus, expression of p75^NTR^ is a common event in GBM. To demonstrate that the presence of p75^NTR^ in these patient specimens confers an increased migratory ability, short-term cultures of these samples were analyzed in transwell motility assays. The percentage of cells positive for p75^NTR^ in the original population was determined by immunostaining and compared to the percentage of p75^NTR^-positive cells in the migratory population (i.e., those cells that migrated to the underside of the transwell chamber during the assay). As a positive control for this assay, a mixture of 25% U87p75 cells and 75% U87pcDNA cells were used as input. At completion of the control assay, the migratory population contained approximately 50% p75^NTR^-positive cells (Figure 6C), as expected from initial experiments that demonstrated that p75^NTR^-positive cells migrate at a greater rate than the p75^NTR^-negative cells (Figure 3E and 3F). Similar effects were observed with the glioma patient specimens. The percentage of p75^NTR^-positive cells in the migratory population compared to the original population was increased by 40%–100% (Figure 6C), demonstrating that the p75^NTR^-positive cells within the glioma patient samples are more migratory than the p75^NTR^-negative glioma cells.

### p75^NTR^ Expression Results in Cytoskeletal Rearrangement and Changes in RhoA Activity

**Figure 7:**
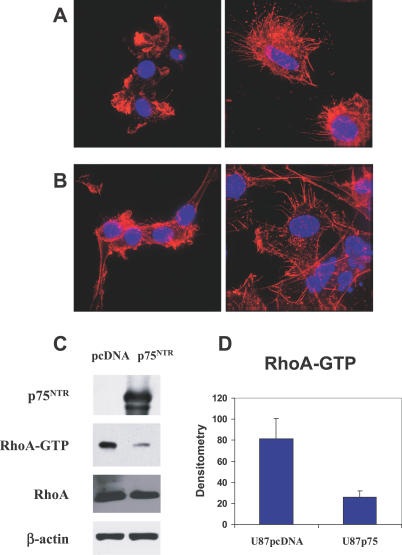
Ectopic Expression of p75 Results in Actin Cytoskeletal Rearrangement and Decreased RhoA Activity (A) Actin staining of tumor (left) and invasive cells (right) shows striking cytoskeletal rearrangement in the invading glioma cells. Actin cytoskeleton was visualized by staining fixed and permeabilized cells with rhodamine phalloidin (red), and cell nuclei were visualized with DAPI (blue). Numerous filamentous protrusions are seen in the invading glioma cells. (B) p75^NTR^ is sufficient to induce cytoskeletal rearrangement of glioma cells. U87pcDNA (left) and U87p75 (right) cells were fixed, permeabilized, and stained with rhodamine phalloidin (red) and DAPI to visualize the nucleus (blue). (C) p75^NTR^ expression results in decreased RhoA activity. RhoA activity was determined in U87pcDNA (pcDNA) and U87p75 (p75^NTR^) by RhoA pulldown assay using a GST fusion protein containing RBD-rhotekin that binds only to activated (GTP-bound) RhoA. Western blots using total cell lysates were performed for p75^NTR^, RhoA, and β-actin (used as a protein loading control). (D) Bar graph shows quantitation of activated RhoA (Rho-GTP) as compared to total RhoA in both U87pcDNA and U87p75. Values shown are the mean ± SEM from four independent experiments.

During the in vitro growth stage of the serial in vivo–selection procedure, we observed that the invasive glioma cells had striking morphological differences to the “tumor” cells. To examine the morphology of these cells, fluorescent staining of the actin cytoskeleton was performed. Staining of the actin cytoskeleton using rhodamine phalloidin revealed cells with numerous filamentous protrusions present only in the invading population (Figure 7A). Similarly, we found that expression of p75^NTR^ alone induced structural rearrangement of the actin cytoskeleton similar to that of the in vivo–selected invasive cells (Figure 7B). Because the small molecular weight GTPase RhoA is a potential downstream readout from p75^NTR^ that may help contribute to the distinct phenotype, we examined the effect of RhoA. Expression studies in HEK293 cells demonstrated that in the absence of ligand, p75^NTR^ constitutively activated Rho, whereas ligand binding leads to a decrease in the levels of active Rho [27]. In addition, Gehler et al. [49] have shown that neurotrophin-bound p75^NTR^ induces growth cone filopodia through the modulation of RhoA and that neurotrophin binding is necessary and sufficient to regulate filopodia dynamics. We found that concomitant with the changes in actin cytoskeleton, cells expressing p75^NTR^ had reduced RhoA activity (Figure 7C and 7D).

## Discussion

Human malignant gliomas are highly invasive tumors. This highly invasive nature associates theses tumors with an extremely poor prognosis owing to recurrence of the tumor outside the margin of therapeutic resection [50]. Invasion of glioma cells into the normal surrounding brain requires changes that make these cells distinct from their noninvasive counterparts. Specifically, these glioma cells activate a number of coordinate cellular programs that involve the regulation of many molecules, including adhesion molecules, extracellular matrix constituents, proteases, cytoskeleton components, and signaling molecules. Altered regulation of any of these constituents may lead to changes in glioma cell migration and invasion. Although numerous molecules have been implicated in the migration and invasion of gliomas, what triggers glioma cells to leave the main tumor mass and invade into the normal brain is not well understood. To this end, we have developed a serial in vivo–selection paradigm to isolate highly invasive glioma cells from a human glioma cell line that is noninvasive in xenotransplantation models. A similar approach has been used to successfully assess the global gene expression profile of both melanoma and breast cancer metastasis [7,8]. Using this strategy, we identified and verified genes that were up-regulated in the invading glioma cells. One of the most differentially expressed genes encodes the neurotrophin receptor p75^NTR^ that is the focus of this study. We provide the first evidence both in vitro and in vivo that p75^NTR^ is a major mediator of glioma migration and invasion.

In recent years, there has been a growing importance of the neurotrophin signaling axis in cancer. Specifically, there is increasing evidence that the neurotrophic receptor tyrosine kinase TrkB, sometimes in conjunction with its primary ligand BDNF, is over-expressed in a variety of human cancers, ranging from neuroblastomas to pancreatic ductal adenocarcinomas [51–55]. Here, we present data that the pan-neurotrophin receptor p75^NTR^ is expressed in malignant glioma and is a major contributor to their highly invasive nature. Although a universal role for p75^NTR^ in cancer has not been established, recent studies implicate p75^NTR^ in the metastatic progression of melanoma, and specifically in those tumors that metastasize to the brain [43,46,56]. Conversely, p75^NTR^ expression has been linked to the progression of prostate cancer, but in this cancer, p75^NTR^, which is expressed in normal prostate epithelia, is lost upon transformation [45]. The divergence observed in the tumor progression of these two distinct tumors can likely be explained by the presence of Trk. In prostrate tumor cells, Trk expression is retained and mediates proliferation [42,57], whereas p75^NTR^-induced invasion in melanoma is independent of Trk expression [58]. Thus the recurring theme emerges that p75^NTR^ function is cell-type specific (even in cancer) and must be independently determined for each cellular context. Here, we have shown that p75^NTR^-induced glioma invasion is also Trk independent with neither mRNA nor protein for the Trk receptors expressed by the invading glioma cells. Further supporting the Trk independence of p75^NTR^-mediated glioma invasion is the finding that treatment of the invasive cells with cleavage-resistant pro-NGF (which cannot bind Trk; Figure S2) also enhanced the migration of invading glioma cells.

Tumor cells can survive by means of an autostimulatory (autocrine) signaling loop, such as that mediated by TrkB and BDNF, or through a paracrine cross-communication with their environment. In brain metastatic melanoma, normal brain tissue adjacent to the melanoma displays increased neurotrophin expression [56], making it tempting to speculate that the metastatic melanoma uses the neurotrophin-rich nervous system as a paracrine mediator of invasion. It has similarly not escaped our attention that the neurotrophin environment of the brain may provide an extremely advantageous milieu for an invading glioma cell. Our data show that the p75^NTR^-expressing glioma cells are ligand responsive and may therefore use neurotrophins available in the brain environment to their advantage. In addition, we show that the invasive nature of glioma cells expressing p75^NTR^ is negated when these cells express mutant p75^NTR^ receptors that no longer bind to neurotrophin.

The concept of p75^NTR^ playing a role in migration is not unprecedented. Neural crest cells, the most extensively studied population of migrating cells in the nervous system, express p75^NTR^ even before they commit to any cell differentiation lineage [59]. In addition, Anton et al. [35] showed that stimulation of the p75^NTR^ by NGF allowed Schwann cells to migrate on peripheral nerves, and examination of p75^−/−^ mice showed severe impairment of Schwann cell migration, with no response to NGF [26]. More recently, the Hempstead laboratory [46] has shown that activation of p75^NTR^ with NGF or pro-NGF (the unprocessed, precursor form of NGF) caused migration of melanoma cells and increased expression of p75^NTR^ correlated with advanced stages and invasive potential of melanoma brain metastasis [60]. At present, the underlying mechanism of p75-induced migration of melanoma cells is not understood; however, p75^NTR^ has been shown to interact with the actin cytoskeleton [46]. The small GTPase RhoA is a downstream effector of p75^NTR^ [27,28]. The capability of p75^NTR^ to modulate the activity of RhoA provides a reasonable explanation as to how p75^NTR^ regulation might result in changes in cellular architecture of glioma cells. We found that concomitant with increased glioma invasion, glioma cells expressing p75^NTR^ showed reduced RhoA activity and striking actin rearrangement.

Previous molecular characterization has defined genetic changes between low-grade and high-grade glioma [61–67]. In addition, molecular signatures of glioblastoma subtypes have been identified, including profiles of primary and secondary glioblastoma subgroups [68–70]. On the other hand, very little is known with respect to the transcriptional profiles of invading glioma cells. Studies have been performed using laser capture microdissection in patient specimens to collect the invasive cells and the cells from the main tumor mass. Although this approach has been used successfully to identify invasion-related genes [9], these experiments make the assumption that the invasive cells at the leading edge of the tumor have distinct profiles from the main tumor mass and that only tumor cells at the invading edge express genes important for migration and invasion. Yet, within the highly heterogeneous environment of a glioblastoma, in which there are many hypoxic and necrotic regions, it would be easy to envision that tumor cells experiencing oxidative stress would activate mechanisms enabling them to move to a more favorable environment. As such, some genes may not be identified using such an approach. Indeed, our data show that in addition to p75^NTR^-expressing glioma cells at the invasive edge of patient tumors, histological analysis identified p75^NTR^-positive glioma cells in regions of the tumor not adjacent to normal brain parenchyma. An alternative explanation for the appearance of p75^NTR^-positive glioma cells is that p75^NTR^ promotes survival of glioma cells in vivo, though we did not find that p75^NTR^ conferred a survival advantage in vitro. Additionally, reports of “stem-like” cells in brain tumors suggest that brain tumors arise from the transformation of neural stem cells [71–73], and when implanted into the brains of SCID mice, these cells form highly invasive tumors [16,74,75]. Whether these brain tumor stem cells express p75^NTR^ is an important question for future studies, especially given that nestin-positive, p75^NTR^-positive cells have been identified in the subventricular zone of the adult brain [76].

Identification of key regulatory proteins of glioma invasion is extremely important clinically because this will be used to provide therapeutically relevant targets to prevent malignant glioma recurrence at the invasive margin of gliomas [4]. Herein, we present the first evidence that p75^NTR^ is important in glioma migration, and the mere expression of p75^NTR^ is sufficient to impart a dramatic invasive behavior on genetically distinct glioblastomas. Because p75^NTR^ has also been implicated in the progression of melanoma, and specifically in those tumors that metastasize to the brain [33,46,77], therapies that target p75^NTR^, p75^NTR^ downstream effectors, or their ligands may not only be beneficial for malignant glioma, but may target other metastatic diseases.

## Materials and Methods

### Cell culture.

The human glioma cell line U87 was obtained from the American Type Culture Collection (http://www.atcc.org). The human glioma cell lines U251N and SF767 were kind gifts from V. W. Yong (University of Calgary, Calgary, Alberta, Canada) and M. Berens (Barrow Neurological Institute, Phoenix, Arizona, United States), respectively. All cells were maintained in complete medium (Dulbecco's Modified Eagle's Medium [DMEM] F12 supplemented with 10% heat-inactivated fetal bovine serum, 1% antibiotic/antimycotic, 0.1 mM nonessential amino acids, 2 mM L-glutamine and 1 mM sodium pyruvate (GIBCO BRL, http://www.invitrogen.com)] at 37 °C in a humidified 5% CO_2_ incubator. Cells were passaged by harvesting with trypsin when they reached 80%–90% confluence. Stable transfectants of U87 and SF767 cells were maintained in the same medium, with the addition of 400 μg/ml G418 or 200 μg/ml hygromycin, respectively (Invitrogen, http://www.invitrogen.com).

### Generation of plasmids.

The GFP expression vector was pGFP-N1 from Clontech (http://www.clontech.com). The human p75^NTR^ expression vector was constructed as described previously [46]. The expression plasmids containing the p75^NTR^ mutants were constructed either by subcloning of PCR fragments containing the desired p75^NTR^ sequences (for p75CRD^130^ construct) or by PCR-based site-directed mutagenesis (for the p75CRD^105^ constructs). Primers used for the construction of the mutants were: p75CRD^105^ primers (sense: 5′-CGG GCT CGG GCC GCT CGA GCG GCC TCG TGT TC–3′; antisense: 5′-GAA CAC GAG GCC GCT CGA GCG GCC CGA GCC CG–3′) and template p75WT [46]; p75CRD^130^ primers (sense: 5′- GAA GAT CTC CAA GGA GGC ATG CCC CAC AGG CC–3′; antisense: 5′-CTC ACT ATA GGT CGA CCG GAA TTC G– 3′) and template pT3/T7-p75. The original templates were from B. Hempstead (p75WT; Cornell University Medical College) and M. Chao (pT3/T7-p75; New York University School of Medicine, New York, New York, United States). The sequences of all the mutant expression plasmids were confirmed prior to stable transfection. The p75^NTR^-specific siRNA expression vector was constructed by ligating a double-stranded hairpin oligonucleotide: 5′-GAT CCG AGG ATC GGA GGC TTG TCA TTC AAG AGA TGA CAA GCC TCC GAT CCT CTT TTT TGG AAA-3′, containing a p75^NTR^-specific siRNA sequence (underlined), into the pSilencer 2.1-U6 hygro vector (Ambion, http://www.ambion.com). The negative control pSilencer vector, containing a random siRNA with limited homology to any known human, mouse, or rat sequences, was obtained from Ambion.

### Transfection of glioma cell lines.

Cells to be transfected were seeded at 2 × 10^5^ cells/well of a six-well plate and incubated at 37 °C overnight in complete media. Vector DNA was introduced to the cells using FuGENE 6 transfection reagent (Roche Diagnostic, http://www.roche.com) according to the manufacturer's instructions. The cells were then incubated at 37 °C overnight; and the following day, the medium was changed to fresh complete medium containing an antibiotic (concentration determined by toxicity curve for cell line) to select for those cells that had taken up the vector. The cells were then grown under antibiotic selection until the wells were confluent. For GFP transfection, transfected cells were identified by fluorescent microscopy and GFP expression of greater than 95% was obtained by fluorescence-activated cell sorting. For p75^NTR^, p75CRD^105^, p75CRD^130^, and p75^NTR^-siRNA transfection, transfected cells were identified by RT-PCR and Western blot.

### Animals.

Six- to 8-wk-old female SCID mice were purchased from Charles River Laboratories (http://www.criver.com). The animals were housed in groups of three to five, maintained on a 12-h light/dark schedule with a temperature of 22 °C ± 1 °C and a relative humidity of 50% ± 5%. Food and water were available ad libitum. All procedures were reviewed and approved by the University of Calgary Animal Care Committee.

### In vivo selection of invasive glioma cells.

Actively growing U87 cells expressing GFP and neomycin resistance genes (U87GFP) were harvested by trypsinization, washed, and resuspended in sterile PBS (137 mM NaCl, 8.1 mM Na_2_HPO_4_, 2.68 mM KCl, and 1.47 mM KH_2_PO_4_ [pH 7.5]). These cells were implanted intracerebrally into the right putamen of SCID mice (1 × 10^5^ cells/mouse) at a depth of 3 mm through a scalp incision and a 0.5-mm burr hole made 1.5–2 mm right of the midline and 0.5–1 mm posterior to the coronal suture. Stereotactic techniques were described previously [77]. Tumor formation was allowed to proceed for 21+ days, depending on the health of the mouse and the type of cells injected. The mice were then sacrificed and the brain examined using fluorescence. The brain was divided in half coronally; one half was used for frozen sections and the other used for tissue culture. For tissue culture, the hemisphere containing the main tumor mass was separated from the contralateral hemisphere, and the two pieces were treated individually. The tissue was minced into small pieces and dissociated with trypsin and DNase I at 37 °C. The tissue suspension was then forced through a 100-μm mesh, and the resulting cell suspension was centrifuged and resuspended in complete medium containing 400 μg/ml G418 to select for the GFP-transfected tumor cells. Cells obtained from the tumor mass were labeled as “tumor” cells, and those from the contralateral hemisphere were labeled as “invasive” cells. Tumor and invasive cells were then reinoculated into SCID mice, and the procedure was repeated.

### RT-PCR.

Total cellular RNA was extracted from subconfluent cells using Trizol Reagent (Invitrogen) and DNase-treated using DNA-free (Ambion). The reverse transcription reaction took place in a buffer of 10 mM Tris-HCl (pH 9.0), 50 mM KCl, and 1.5 mM MgCl_2_, and contained 3 μg of total RNA, 25 units of RNAguard RNase inhibitor, 1 mM each of deoxynucleoside triphosphates, 100 ng of pd(N)_6_ random hexanucleotide primers (Amersham Biosciences, http://www.amersham.com), and 200 units of Superscript II reverse transcriptase (Invitrogen). The PCR amplification reaction was carried out in the same buffer and contained 1 μl of the cDNA synthesis reaction, 80 μM each of deoxynucleoside triphosphates, 1 unit of Taq DNA polymerase (Amersham Biosciences), and 0.1 μM each of p75^NTR^-specific primers (forward: 5′-CGT ATT CCG ACG AGG CCA ACC-3′; reverse: 5′-CCA CAA GGC CCA CAA CCA CAG C-3′), p75CRD^105^-specific primers (forward: 5′-CGG GCT CGG GCC GCT CGA GCG GCC TCG TGT TC-3′; reverse primer: 5′-GAA CAC GAG GCC GCT CGA GCG GCC CGA GCC CG-3′), p75CRD^130^-specific primers (forward: 5′-GAA GAT CTC CAA GGA GGC ATG CCC CAC AGG CC-3′; reverse primer: 5′-CTC ACT ATA GGT CGA CCG GAA TTC G-3′), or 0.2 μM each of GAPDH-specific primers (forward primer: 5′-CGG AGT CAA CGG ATT TGG TCG TAT-3′; reverse primer: 5′-AGC CTT CTC CAT GGT GGT GAA GAC-3′). The amplification consisted of 35 cycles of 45 s at 94 °C, 30 s at 63 °C, and 45 s at 72 °C, followed by a 7-min extension at 72 °C after the last cycle. The reaction products were then resolved on a 1% agarose gel containing ethidium bromide.

### Western blotting.

Total cellular lysates were obtained by gentle rocking in lysis buffer (20 mM Tris [pH 8.0], 137.5 mM NaCl, 10% glycerol, 1% Nonidet P-40, 25 μg/ml aprotinin, 10 μg/ml leupeptin, 3 mM sodium orthovanadate, 1 mM PMSF) at 4 °C. Protein extracts of human glioma biopsies were obtained by immersing the samples in ice-cold extraction buffer (50 mM Tris [pH 7.6], 200 mM NaCl, 10 mM CaCl_2_, 1% Triton X-100) followed by homogenization on ice. Cellular debris was removed by centrifugation, and protein quantification was performed using the bicinchoninic acid (BCA) assay (Pierce Biotechnology, http://www.piercenet.com). Proteins were resolved on 10% SDS-PAGE gels, and Western blots were performed using the following primary antibodies: rabbit polyclonal anti-human p75^NTR^ (Promega, http://www.promega.com), goat polyclonal anti-pyruvate kinase (Chemicon, http://www.chemicon.com), mouse monoclonal anti-neurofilament, 70 kDa (Chemicon), mouse monoclonal anti-glial fibrillary acid protein (ChemiconA), or mouse monoclonal anti-β-tubulin (Sigma-Aldrich, http://www.sigmaaldrich.com). The appropriate HRP-conjugated secondary antibody (Pierce Biotechnology) was used and visualized using enhanced chemiluminescence (Amersham Biosciences).

### Flow cytometric analysis of p75^NTR^ expression.

Cells were collected using Puck's EDTA at 37 °C and then washed in PBS containing 1 mM EDTA (PBS/EDTA). Cells were then treated with monoclonal anti-p75^NTR^, clone ME20.4 (which recognizes the extracellular domain; Upstate Biotechnology, http://www.upstate.com), diluted 1:250 in PBS/EDTA for 30 min on ice. The negative control sample was incubated in only PBS/EDTA. After washing with PBS/EDTA, cells were treated with Alexa 488–conjugated goat anti-mouse IgG (Invitrogen Molecular Probes, http://probes.invitrogen.com) diluted 1:500 in PBS/EDTA for 30 min on ice. Cells were then washed with PBS/EDTA, resuspended in PBS/EDTA, and analyzed on a FACScan flow cytometer (Becton, Dickinson and Company, http://www.bdbiosciences.com).

### Enzyme-linked immunosorbent assay.

Cells were allowed to condition the medium for 5 d. The conditioned medium was then collected, centrifuged, and filtered through a 0.2-μm syringe filter (VWR International, http://www.vwr.com). The remaining cells were washed with ice-cold PBS, and total cellular lysates were extracted as described for Western blot. Protein quantification was performed using the BCA assay (Pierce Biotechnology) and BDNF enzyme-linked immunosorbent assays (ELISAs) (R&D Systems, http://www.rndsystems.com) were performed as per the company protocol. Briefly, MaxiSorp ELISA plates (Nalge Nunc International, http://www.nalgenunc.com) were coated with monoclonal anti-human BDNF (R&D Systems), nonspecific binding was blocked, and serial dilutions of recombinant human BDNF (Sigma-Aldrich), equal volumes of conditioned medium, or equal quantities of lysate were added. Bound antigen was detected using the corresponding biotinylated antibody, streptavidin HRP, and a tetramethylbenzidine substrate (R&D Systems).

### Circular monolayer migration assay.

Migration assays were performed using a microliter-scale radial monolayer migration assay as described by Berens et al. [78]. Briefly, ten-well Teflon-masked microscope slides were coated with 20 μg/ml laminin. Cells were seeded through a cell sedimentation manifold (Creative Scientific Methods, http://www.cre8ive-sci.com) at 2,000 cells/well to establish a circular 1-mm diameter confluent monolayer. Once the sedimentation manifolds were removed, cells were given complete medium containing the appropriate treatment. A digital image of the cells was taken (before migration = 0 h) using a Zeiss Axiovert 200M inverted fluorescent microscope (Carl Zeiss, http://www.zeiss.com). The cells were then incubated in a humidified chamber at 37 °C and 5% CO_2_, and a second digital image was taken 48 h later. Best-fit circles were drawn around the area covered by the cells at the 0-h and 48-h time points and the actual cell area determined using Axiovision 4.2 imaging software (Carl Zeiss). Quantitative migration scores were calculated as the increase in the area covered by the cells beyond the initial area of the cells.

### In vitro invasion assay.

Matrigel (BD Bioscience, Mississauga, Ontario, Canada) was diluted with two parts of cold serum-free medium, layered onto an 8-μm pore-size transwell chamber (BD Bioscience, http://www.bdbiosciences.com), and incubated at room temperature for 1 h. The wells were then rinsed with serum-free medium. The coated chambers were placed into the wells of a 24-well tissue culture plate containing 500 μl of media with or without the desired treatment. Serum-starved cells (2.5 × 10^4^) were seeded into each chamber, in a volume of 500 μl of the same medium contained in the bottom of the well, and incubated at 37 °C for 48 h. The medium was then removed from the chambers and cells scraped off the top of the membrane using a PBS-soaked cotton-tipped swab. Cells were fixed to the bottom of the chamber with methanol, stained in hematoxylin, and mounted on slides. Invasion was quantified by counting the stained cells adherent to the lower side of the membranes in ten fields (at 10× magnification) for each of three chambers for each condition.

### In vivo studies of p75^NTR^ overexpression in an intracranial glioma model.

Actively growing U87pcDNA, U87p75, U87CRD^105^, U87CRD^130^, U251NpcDNA, and U251Np75 cells were implanted intracerebrally into SCID mice as described previously [77]. Mice were sacrificed weekly from day 14–42. At each time point, the brains were removed, frozen in OCT compound (Tissue-Tek; Electron Microscopy Sciences, http://www.emsdiasum.com), and cryosectioned for examination by immunohistochemistry.

### Immunohistochemistry.

Frozen sections were air dried at room temperature, fixed with cold acetone, and then rinsed with PBS. Paraffin sections were dewaxed and rehydrated using a xylene/ethanol series followed by rinsing with PBS. Endogenous peroxidases in the sections were inactivated with 0.075% H_2_O_2_/methanol, and nonspecific binding was blocked with 10% normal goat serum in PBS. The sections were then incubated with rabbit polyclonal anti-human p75^NTR^ (Promega, http://www.promega.com) or mouse monoclonal anti-human nuclei (Chemicon) in blocking buffer overnight at 4 °C. Following washing with PBS, the appropriate biotinylated secondary antibody (Vector Laboratories, http://www.vectorlabs.com) was applied. Avidin-biotin peroxidase complexes were then formed using the VECTASTAIN Elite ABC kit (Vector Laboratories) and detected by addition of SIGMAFAST DAB (3,3′-diaminobenzidine tetrahydrochloride) (Sigma-Aldrich). The SIGMAFAST DAB was converted to a brown reaction product by the peroxidase. Hematoxylin (for paraffin sections) and toluidine blue (for frozen sections) were used as nuclear counterstains. Sections were then dehydrated in an ethanol/xylene series and mounted with Entellan (Electron Microscopy Sciences).

### Immunocytochemistry.

Coverslips were coated with a Collagen I (3 mg/ml; Vitrogen 100; Cohesion Technologies, http://www.cohesiontech.com) and incubated overnight at 37 °C. Excess collagen solution was aspirated, and cells were plated at 2 × 10^5^/mL in DMEM culture medium (DMEM with 10% FBS, 6 mM L-glutamine, 100 μM nonessential amino acids, 1 mM sodium pyruvate, 400 μg/ml G418) and allowed to equilibrate overnight at 37 °C, 5% CO_2_. Coverslips were then rinsed twice with PBS, fixed in 3.7% formaldehyde diluted in PBS for 10 min, and rinsed twice with PBS. Unpolymerized actin was extracted for 3 min in CSK buffer (10 mM MES [pH 6.1], 138 mM KCl, 3 mM MgCl_2_, 2 mM EGTA, 320 mM sucrose, 0.1% Triton X-100) followed by two rinses with PBS. Alexa Fluor 568 phalloidin (Invitrogen) was diluted 1:40 in 1% BSA/PBS and 200 μl of this solution was added to each coverslip for 20 min at room temperature. Coverslips were rinsed twice with PBS, counterstained with a 500 nM solution of DAPI for 3 min, mounted in glycerol, and imaged with an Olympus IX70 Delta Vision RT Microscope (http://www.olympus.co.jp/en/) and the SoftWoRx software package.

### Tumor tissue.

Tumor and normal tissues were obtained from the Canadian Brain Tumor Tissue Bank in London, Ontario, and Foothills Hospital, Calgary, Alberta. Briefly, tissue was taken during surgery while patients were under a general anesthetic, and was placed immediately in liquid nitrogen and stored at −80 °C or placed in culture medium for establishment of short-term cultures. An institutional ethics board approved the collection and use of all of the surgical tissue used, and all of the patients gave signed informed consent. The following tissues were studied: 20 GBMs, eight anaplastic astrocytomas, one anaplastic oligodendroglioma, five astrocytomas, five mixed oligoastrocytomas, one oligodendroglioma, and five controls obtained during nontumor brain surgery.

### Short-term culture of primary human glioma cells.

Operative samples of human gliomas were obtained during brain tumor surgery and transported to the laboratory in culture medium. Short-term cultures were then established. Briefly, necrotic and connective tissue and any blood clots were removed using forceps, and the remaining tissue was washed in PBS and cut into pieces of approximately 1 mm^2^. The tissue was then incubated for 30 min at 37 °C in an enzyme cocktail of trypsin (0.25%) and DNase I (10 μg/ml) in PBS. The digested tissue was strained through a 100-μm mesh and washed with PBS. The cells were then pelleted and washed with DMEM-F12 media. Following lysis of red blood cells, the remaining cells were washed with PBS, pelleted, resuspended in complete media containing 20% FBS, and plated.

### Transwell motility assay of primary human glioma cells.

Primary human glioma cells cultured for less than 3 wk were “serum-starved” by incubating them in medium containing only 1% FBS for 2 h at 37 °C and 5% CO_2_. Cells were then released from the culture dish using Puck's EDTA (1 mM EDTA, 10 mM HEPES, 5 mM KCl, 140 mM NaCl, 4 mM NaHCO_3_, and 6 mM dextrose [pH 7.3]) at 37 °C. Cells (2.5 × 10^4^) suspended in 1% FBS medium were plated in eight-well chamber slides (Nunc) and transwell chambers (Costar) coated with 20 μg/ml laminin. Medium containing 1% FBS was placed below the chamber. The cells were incubated at 37 °C and 5% CO_2_ for 6 h. The medium was then removed from the chambers, and cells were fixed with 4% paraformaldehyde. The cells were then stained for human p75^NTR^, as described for immunohistochemical staining, and counterstained with hematoxylin. In the transwell chamber, cells that did not migrate were scraped off the top of the membrane using a cotton-tipped swab. Migration was quantified by counting the p75^NTR^-positive (brown) and p75^NTR^-negative (blue) cells in the original population (on the slide) or in the migratory population (adherent to the under side of the transwell membrane) in five fields (at 20× magnification) for each of four chambers.

## Supporting Information

Figure S1 In Vivo–Selected U251 Invasive Cells Express p75^NTR^
U251 human glioma GFP-expressing cell line was implanted into the brains of SCID mice; and 4–6 wk later, the mice were sacrificed. The ipsilateral side of the brain (containing a grossly visible tumor) was separated from the contralateral side (containing only isolated invasive glioma cells) and both were grown in culture. These noninvasive (tumor [U251T]) and highly invasive (invasive [U251R]) cells were reimplanted and the process repeated to select for increasingly noninvasive or invasive glioma cells. Through the serial in vivo selection, highly invasive U251 glioma cells were isolated. Western-blot analysis of p75^NTR^ expression in tumor and invasive U251 human glioma cells shows a dramatic increase in endogenous p75^NTR^ expression in the U251 invasive cells as compared to the U251 tumor cells. U87 tumor (U87T) and invasive (U87R) cells were used for comparison (A). The invasive U251 cells, which endogenously express p75^NTR^, showed a significant increase in migration (B) and invasion (C) compared to U251 tumor cells. Double asterisks(∗∗) indicate *p* < 0.001 (tumor vs. invasive, paired *t*-test). Values shown are the mean ± standard error of the mean (SEM) from three independent experiments.
(136 KB TIF)

Figure S2 Invasive Glioma Cells Expressing p75^NTR^ Increase Migration in Response to Cleavage-Resistant Form of pro-NGF Treatment of invasive cells with a cleavage-resistant form of pro-NGF significantly increased migration at concentrations as low as 1 ng/ml. Values shown are the mean ± SEM for a single experiment. Similar results were seen in three independent experiments Double asterisks (**) indicate *p* < 0.01, and triple asterisks (***) indicate *p* < 0.001 versus control (one-way analysis of variance [ANOVA] with Bonferroni post-test). Statistics were done on the experiment shown.
(58 KB TIF)

Figure S3 Autocrine BDNF Is Bound to the Cell Surface of p75^NTR^-Expressing U87 Cells BDNF expression was confirmed by RT-PCR (A). The amount of BDNF protein contained in the conditioned medium (B) or cell associated before and after treatment with a cell surface-stripping reagent (C) was measured by ELISA in cells expressing either empty vector (pcDNA) or a p75^NTR^-expression vector (p75), U87 pcDNA and U87p75. Values shown are the mean ± SEM from a single experiment; triple asterisks (***) indicate *p* < 0.001 U87p75 compared to pcDNA; triple pluses (+++) indicate *p* < 0.001 U87p75 before and after cell surface stripping. Similar results were seen in three independent experiments.
(153 KB TIF)

Figure S4 Down-Regulation of p75^NTR^ Expression Using RNAi Decreases Migration and Invasion of In Vivo–Selected U87 Invasive Glioma Cells (A) RT-PCR (GAPDH used as a loading control) and (B) Western blot (pyruvate kinase used as a loading control) confirm down-regulation of p75^NTR^ in the in vivo–selected invasive glioma cells, transiently transfected with a p75^NTR^-specific siRNA. (C) Down-regulation of p75^NTR^ levels by siRNA in the in vivo–selected invasive glioma cells significantly reduces their circular migration and transwell invasion. Values shown are the mean ± SEM from four independent experiments. Double asterisks (**) indicate *p* < 0.01 versus random siRNA transfected cells (paired *t*-test).
(136 KB TIF)

Figure S5 U87p75 Glioma Cells Are Found Substantial Distances from the Main Tumor Mass To further illustrate the invasive nature of the p75^NTR^-expressing glioma cells, serial sections of brains from animals implanted with either pcDNA or p75^NTR^-expressing U87 glioma cells were stained using a human nuclear-specific antibody. U87p75 glioma cells were found substantial distances from the main tumor mass; right panel shows whole-brain sections posterior to the main tumor. (First two panels are the same as Figure 4).
(1.1 MB TIF)

## Abbreviations

ANOVA: analysis of variance
BDNF: brain-derived neurotrophic factor
CRD: cysteine-rich domain
ELISA: enzyme-linked immunosorbent assay
GBM: glioblastoma multiforme
GFP: green fluorescent protein
NGF: nerve growth factor
p75^NTR^: p75 neurotrophin receptor
pro-NGF: proform of NGF
RT-PCR: real-time polymerase chain reaction
SCID;: severe combined immunodeficiency
siRNA: small interfering ribonucleic acid
Trk: tropomysin receptor kinase
